# Colchicine-induced degeneration of the micronucleus during conjugation in *Tetrahymena*

**DOI:** 10.1242/bio.20147708

**Published:** 2014-04-11

**Authors:** Pin-Fang Chen, Sita Singhal, Daniel Bushyhead, Sarabeth Broder-Fingert, Jason Wolfe

**Affiliations:** Department of Biology, Wesleyan University, Middletown, CT 06459, USA; * Present address: Department of Genetics and Developmental Biology, University of Connecticut Health Center, Farmington, CT 06030, USA.; ‡ Present address: Internal Medicine Residency Program, University of Connecticut Health Center, Farmington, CT 06030, USA.; § Present address: School of Medicine, University of Washington, Seattle, WA 98195, USA.; ¶ Present address: Department of Pediatrics, Massachusetts General Hospital, Boston, MA 02114, USA.

**Keywords:** Apoptosis, Autophagy, Ciliate, Nuclear morphogenesis, Meiosis, Micronucleus, *Tetrahymena*

## Abstract

One of the most dramatic examples of nuclear morphogenesis occurs during conjugation in *Tetrahymena* when the micronucleus elongates to a size longer than the cell itself. After contraction to a spherical shape, the nucleus moves directly to chromosome separation in the first meiotic division. Here we investigate the consequences of interrupting the elongation process. Colchicine, a microtubule inhibitor, caused retraction of elongated structures. With time, cells began to lose their micronuclei, and by five hours more than half of the paired cells had at least one cell missing a micronucleus. After reversing the colchicine block, existing micronuclei did not undergo elongation again, nor did meiosis occur. These observations indicate that micronuclear elongation is critical to subsequent meiotic division. Further, nuclear elimination occurs, which could be due to meiotic failure or possibly a problem downstream from meiosis. An analysis of the process of colchicine-induced micronuclear degeneration indicated that it was regulated by a caspase-dependent mechanism, characteristic of apoptosis, and then resorbed by a lysosome-dependent autophagic mechanism. Amicronucleate cells failed to grow when returned to nutrient medium, likely because of a lesion in the post-conjugation reconstruction of a functioning oral apparatus. The ease by which a large number of nuclei are induced to “self-destruct” may make this system useful in investigating the link between colchicine treatment and nuclear death in *Tetrahymena*, and in investigating how nuclear death could be regulated in living cells more generally. Finally, we note that this phenomenon might relate to the evolution of amicronucleate species of *Tetrahymena*.

## INTRODUCTION

The size and shape of a nucleus is a phenotypic characteristic of the cell in which it is located (Georgatos, 1994). Correct morphogenesis of nuclei can be critical for development, such as in the differentiation of sperm cells (Dadoune, 1995) and during cellularization of the cortex in *Drosophila* embryos (Pilot et al., 2006). Nuclear change shape also takes place during the epithelia-to-mesenchyme transition in tumor cells, and may contribute to metastasis (Thiery et al., 2009; Yao et al., 2011).

One of the most dramatic instances of nuclear shape change occurs during conjugation in *Tetrahymena*, when shortly after cell pairing, the small (∼1 µM) micronucleus rapidly elongates reaching a 50:1 length to width ratio (Ray, 1956b). *Tetrahymena*, like other ciliated protozoa, contains two types of nuclei, a large, highly polyploid macronucleus that regulates the ongoing metabolism of the cell, and divides by amitosis, and a smaller diploid micronucleus that divides by mitosis with each cell doubling (Orias et al., 2011; Karrer, 2012). The micronucleus is also capable of meiosis, the first step of which is its elongation (Ray, 1956a; Sugai and Hiwatashi, 1974). During vegetative growth gene expression is not detected from the micronucleus (Mayo and Orias, 1981), and it is metabolically inactive (Gorovsky and Woodard, 1969). However, based on autoradiography during conjugation, a limited amount of micronuclear RNA synthesis may occur briefly just as the structure begins to elongate (Sugai and Hiwatashi, 1974).

Chromatin is organized in the elongated micronucleus with telomeres located at one end of the elongated structure (Loidl and Scherthan, 2004), and centromeres at the other (Loidl and Mochizuki, 2009), creating a classical meiotic chromosomal bouquet. It has been suggested that, in the absence of synaptonemal complexes (Wolfe et al., 1976; Loidl and Scherthan, 2004), micronuclear elongation may provide conditions for homologue alignment during the first meiotic division (Loidl and Scherthan, 2004).

From earlier studies it is known that micronuclear elongation is a microtubule-dependent process (Wolfe et al., 1976; Wolfe, 1978). Microtubules appear inside the nucleus at the time that elongation begins, and they grow in length, as the micronucleus gets longer. They are attached at their origin to a dense body, likely a microtubule-organizing center, and occupy a position subjacent to the nuclear envelope, forming a cage-like structure around the chromatin. The process of elongation is dependent on microtubule polymerization, as was demonstrated by the effect of the microtubule inhibitor nocadazole, which prevented elongation and caused rapid collapse of partially or fully elongated micronuclei (Wolfe, 1978; Kaczanowski et al., 1985).

In this study we employed another microtubule inhibitor, colchicine, to analyze the consequences to the cell when micronuclear elongation is prevented or interrupted. We found, as had been shown by Kazcownowski et al. using nocodazole (Kaczanowski et al., 1985), that meiosis is irreversibly blocked. But we also discovered that some of the collapsed micronuclei – in fact a large proportion (over 60% within 5 hours) – disappeared from the cell.

This was a startling finding. Although we had been studying programmed macronuclear elimination during conjugation (Mpoke and Wolfe, 1996; Mpoke and Wolfe, 1997; Lu and Wolfe, 2001; Ejercito and Wolfe, 2003), as well as the degradation of the three post-meiotic haploid nuclei that do not go on to produce gamete nuclei (Santos et al., 2000), we were surprised that nuclear loss could be induced by experimental manipulation. Since nuclear loss in this system occurred reproducibly and in large numbers, it lent itself to further analysis.

In this study we sought to determine the mechanism by which micronuclear elimination occurs. In addition, we investigated the impact on the cell of the loss of its micronucleus by examining what happened to blocked paired cells when they are returned to growth medium.

Previous work from this laboratory and others indicated that an apoptosis-like process regulates the developmentally programmed elimination of the parental macronucleus during conjugation, and that its resorption is under control of autophagy (Mpoke and Wolfe, 1996; Lu and Wolfe, 2001; Ejercito and Wolfe, 2003; Endoh and Kobayashi, 2006; Akematsu and Endoh, 2010; Liu and Yao, 2012). In this study we show that this also applies to colchicine-induced micronuclear degeneration.

## MATERIALS AND METHODS

### Growth and conjugation

Cells of mating types III and VII of *Tetrahymena thermophila* subline B, obtained originally from Norman Williams, were cultured and prepared for conjugation as described previously (Mpoke and Wolfe, 1997). To initiate conjugation cells of complementary mating types that had been starved overnight in 10 mM Tris buffer (pH 7.4) were mixed together in equal volumes in a 60 mM sterile plastic Petri dish. The mixed cells were incubated at 30°C. Pairing was generally observed by one hour after mixing, and typically by three hours about 80% of the cells had formed pairs.

### Cell pairing and nuclear staining

Samples of cells at different stage of conjugation were fixed in an equal volume of 3% paraformaldehyde in PHEM buffer (60 mM PIPES, 25 mM HEPES, 10 mM EGTA, and 2 mM magnesium chloride) with 0.5% Triton X-100. This fixative (P-PHEM-TX) was used specifically to increase microtubule stability and improve staining contrast in fluorescence microscopy. Fixative was stored at room temperature. Nuclei of fixed cells were stained either with 0.1 µg/ml 4,6-diamidino-2-phenylindole (DAPI) or with 0.05 µM Sytox. The latter was used especially for observations with the confocal microscope. Some samples were resuspended in 200 µL NPG (25% glycerol and 1% N-propylgallate in phosphate buffer at pH 7.0) to retard photo-bleaching (Giloh and Sedat, 1982). Cells were observed by fluorescence microscopy using a filter for blue light at a wavelength of 359 nM (for DAPI) or 488 nM (for Sytox). Sample size for quantitative assays consisted of 200 cells (from 100 pairs).

Acidification of degrading nuclei in unfixed, living cells was assayed using two parts of 0.1 mg/ml acridine orange and one part of 1 mg/ml Hoechst 33342 (Mpoke and Wolfe, 1997; Santos et al., 2000). The acridine orange enters acidic compartments and stains them red–orange, while Hoechst 33342 stains DNA blue. Living cells were immobilized, as described previously, to minimize motility and permit photomicrography (Santos et al., 2000).

Freshly shifted down cells of both mating types were incubated in 0.5 mg/mL fluorescent Texas Red Dextran (TRD) to load that marker into lysosomes. After 3 hours of incubation, the cells were washed by centrifugation 5 times with Tris buffer. At the fifth wash, the pellet was brought up to 5 mL, and the cells were permitted to starve overnight. Mating types were mixed as described above, and samples were taken at various time intervals. Cells were observed live with fluorescence microscopy at a wavelength of 543 nM.

### Fluorescence microscopy

Most analyses were made with a Zeiss Axioplan epifluorescence microscope. Micrographs were taken manually with an automatic camera loaded with Fujichrome Sensia II 200 film. Observations were also made with a Zeiss LSM 510 Confocal Microscope. In both cases, editing of micrographs was done with Adobe Photoshop 6.0.

### Reagents

Colchicine, 3 methyladenine, Cytochalasin B, Hoechst 33342 and DAPI were obtained from Sigma–Aldrich Corp., St Louis, MO, USA, zVAD-fmk from R&D Systems, Minneapolis, MN USA, and Sytox and Texas Red Dextran from Molecular Probes, Eugene OR, USA. Proteose Peptone and yeast extract were obtained from Difco, Detroit, MI, USA.

## RESULTS

**Fig. 1.**
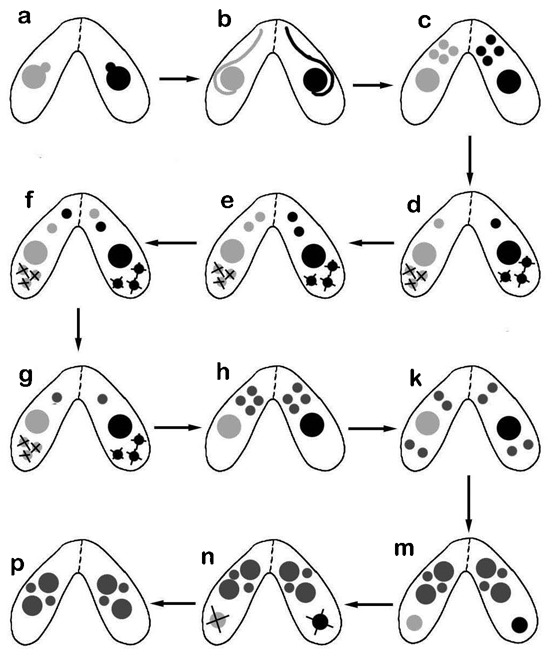
Diagram of the nuclear stages that occur during conjugation of mating types of *Tetrahymena*. The rounded micronucleus moves from its pocket in the macronucleus (a), elongates (b), then contracts and undergoes 2 meiotic divisions forming four haploid products (c). Three haploid nuclei degrade (d) and the remaining haploid nucleus divides by mitosis to form two gametic pronuclei (e). After reciprocal exchange (f), fertilization occurs forming a diploid zygote nucleus (g). The zygote nucleus divides twice mitotically to form four nuclei (h), two of which enlarge to form developing macronuclei and two of which form micronuclei, while the old macronucleus condenses and moves to the posterior end of the cell (k,m). The old macronucleus is then eliminated (n,p).

Fig. 1 is a diagram summarizing nuclear events during conjugation. Shortly after successful cell pairing, which includes adhesion of cells at their anterior ends, followed by cell-membrane fusion when the pores in the conjugation junction are formed (Wolfe, 1985), the micronucleus migrates from an invagination in the macronucleus (Fig. 1a) and then begins to elongate (Fig. 1b). After elongation is completed, the structure contracts and undergoes two meiotic divisions to produce four haploid nuclei (Fig. 1c).

**Fig. 2.**
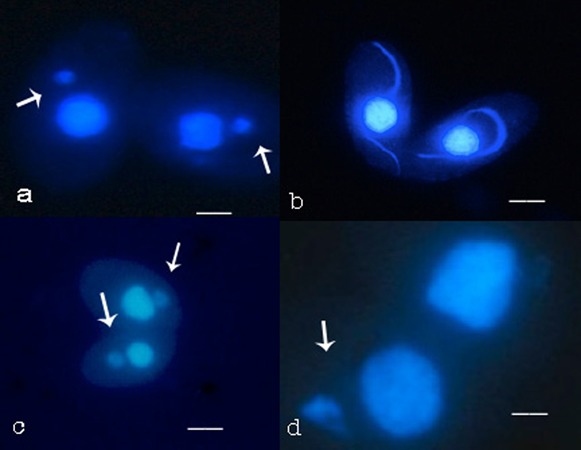
DAPI-stained cells during conjugation. Uninterrupted micronuclear elongation, and elongation reversed with colchicine. Shortly after cell pairing the micronucleus (arrows) migrates slightly from its position in a macronuclear pocket to a cytoplasmic position near the macronucleus (a). It then elongates and curls around the macronucleus (b). Elongated nuclei collapse (arrows) in response to colchicine (c) and may disappear from one (d) or both cells (not shown). Scale bars: 5.7 µm (a), 10 µm (b), 8 µm (c), 3.6 µm (d).

To illustrate these stages in fixed cells stained with DAPI, Fig. 2a shows a pair of conjugating cells whose micronuclei have emerged from the macronucleus but have not yet elongated. Fig. 2b shows a pair with partially elongated micronuclei. By 2.5 hours after mixing starved cells of two different mating types the great majority of paired cells were in various stages of micronuclear elongation. We exposed cells at 2.5 hours to 5 mg/ml of the microtubule inhibitor colchicine. Adding colchicine prior to 2.5 hours had an adverse effect on cell pairing, and was thus avoided. For example, when added at the time of mixing mating types, colchicine reduced pairing by about 90% at 2 hours, relative to control cells (data not shown).

After three hours of exposure to colchicine, at 5.5 hours post-mixing, no elongated micronuclei were visible. Instead, all of the micronuclei had retracted into somewhat sphere-shaped structures. Even after full retraction the micronuclei were not perfectly spherical, and could often be distinguished from micronuclei that had not yet elongated. Compare micronuclei in Fig. 2c,d to those in Fig. 2a. In addition, micronuclei remained somewhat spherical during five hours of incubation with colchicine, and did not undergo meiotic division.

The surprise observation was that after some time elapsed, some cells in pairs were observed to be missing a micronucleus; based on the absence of a DAPI stained structure, the micronucleus was lost (Fig. 2d). Some pairs, as in Fig. 2d, had only one cell missing a micronucleus. In some pairs both cells lost a micronucleus. At early times after treatment with colchicine, the majority of pairs had only one cell missing a micronucleus.

**Fig. 3.**
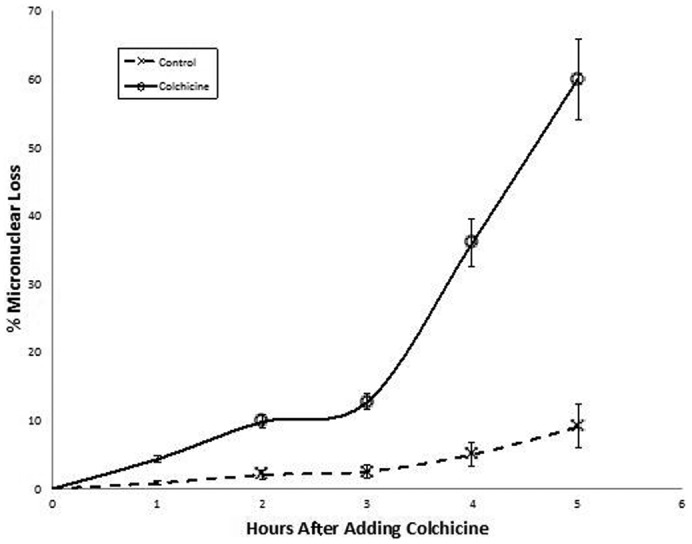
Cells were exposed to 5 mg/ml colchicine at 2½ hours after mixing mating types. Samples were then fixed at hourly time points. Cells in pairs were scored for the presence or absence of a visible micronucleus. These data (open circles) show that the disappearance of the micronucleus increases with time. At 5 hours after adding colchicine micronuclei have been lost in more than half of all individual cells in pairs. Control cells also show some loss of micronuclei (marked ‘x’), which could reflect a spontaneous micronuclear loss, or could indicate error in this visual assay, or both. For this and other graphs, experiments were done in triplicate. Data points show averages, and bars represent range in variation.

Further, the number of cells missing a micronucleus increased with time. To quantify the temporal dynamics of micronuclear elimination, we measured the percentage of paired cells without a micronucleus at hourly intervals after adding colchicine. Fig. 3 shows that micronuclear loss increased with time, rising to more than 60% by 5 hours of exposure (7.5 hours after mixing mating types). Presumably, further loss would occur with increased time, since the curve had not yet leveled off at 5 hours. The delay in nuclear loss between 2 and 3 hours might hint at a 2 population response to colchicine. Fig. 3 also shows some micronuclear loss even in control cells. This could be a measure of spontaneous micronuclear elimination, a measure of error in this visual assay, or a combination of both.

A second surprise was that loss of the micronucleus was not synchronous in pairs, despite cytoplasmic continuity achieved by the conjugation junction. Initially many more pairs had one cell missing a micronucleus while a micronucleus was clearly visible in the other cell (e.g. Fig. 2d). These data indicate that a cytoplasmic factor that can readily pass between the cells of a pair through the conjugation junction is not the apparent trigger for micronuclear elimination. With time, more and more pairs could be seen with both cells lacking a micronucleus. This is consistent with micronuclear elimination being a cell-autonomous, perhaps stochastic, event.

We wondered whether overnight starvation of the cells could have sensitized micronuclei to be resorbed in response to colchicine. However, starved single cells that did not pair did not lose micronuclei in response to colchicine. Therefore, it is not starvation per se that drives micronuclear elimination in response to colchicine. Further, colchicine did not lead to micronuclear loss among vegetative cells in logarithmic growth, although both cell division and micronuclear mitosis were blocked. This indicates that blocking microtubule-dependent nuclear division with colchicine is not the key to micronuclear elimination. Rather, micronuclear elimination in response to colchicine seems to be related specifically to reversing elongation of the micronucleus during conjugation, or to some downstream event after micronuclear elongation.

The first event after normal micronuclear elongation is its contraction followed by meiotic divisions. In control cells at 5.5 hours after mixing mating types, most pairs have already completed meiosis, with 4 (haploid) nuclei visible in addition to the macronucleus (Fig. 1c). However, colchicine-treated cells had only one micronucleus per cell indicating that meiosis was also blocked by colchicine. Since meiosis is a microtubule-dependent event, this result is not unexpected. Therefore, micronuclear loss could result from a failed meiosis. After meiosis in control cells, three haploid products are resorbed, and one persists and divides to produce gamete nuclei (Fig. 1d–f). The nucleus that survives is saved from default elimination by becoming physically associated with the conjugation junction (Numata et al., 1985; Takagi et al., 1991; Gaertig and Fleury, 1992). Therefore, micronuclear loss in response to colchicine could be related to a block of micronuclear elongation, a block of meiosis, or the default result of failure to achieve linkage to the conjugation junction. These three possibilities will be considered more fully in the Discussion.

Close observation of living cells using the vital DNA stain Hoechst 33342 indicated that the micronucleus was not ejected from the cell, as occurs during terminal differentiation of red blood cells in mammals (Skutelsky and Danon, 1967). Instead, the structure was degraded; it got smaller and faded away.

In that regard, colchicine-induced micronuclear degeneration (CIMD) showed similarities to developmentally programmed degradation of the parental macronucleus during conjugation. In both cases the nucleus grows smaller and eventually is no longer visible with DAPI staining, and both are resorbed within the cell. The similarities suggested that the two processes might share a common mechanism.

**Fig. 4.**
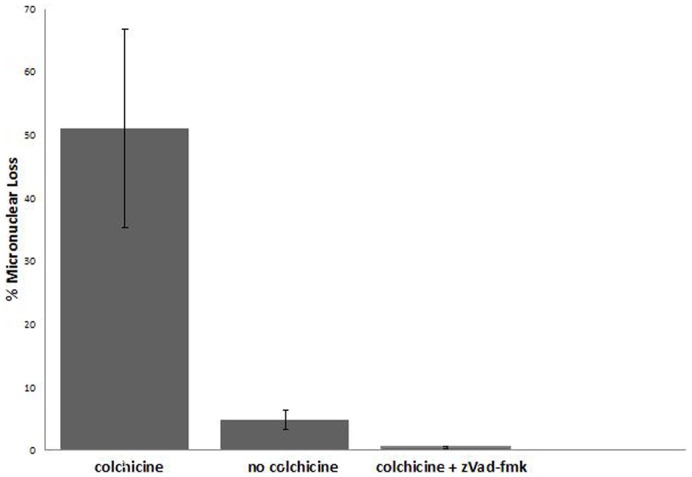
Bar graph showing the effect of zVAD-fmk, a caspase inhibitor, on colchicine-induced micronuclear degeneration. With colchicine alone (5 mg/ml, 2.5–7.5 hours after mixing mating types) about 50 percent of cells in pairs lose micronuclei. As in Fig. 3, even control cells, with no colchicine added, show some micronuclear loss. However, when zVAD is added together with colchicine, micronuclear loss is almost completely inhibited.

We, and others, had shown that an apoptosis-like process, sensitive to inhibitors of caspase activity, regulates developmentally programmed macronuclear elimination (Fig. 1k–p) (Mpoke and Wolfe, 1996; Ejercito and Wolfe, 2003; Kobayashi and Endoh, 2003; Akematsu and Endoh, 2010). We therefore examined whether the caspase inhibitor zVAD-fmk had any effect on CIMD. Fig. 4 shows that compared to paired cells incubated for 5 hours in colchicine alone, zVAD-fmk (1 mM) together with colchicine almost complete negates the effect of colchicine, demonstrating that CIMD is dependent on caspase-like activity. Therefore, like regulated macronuclear degeneration, CIMD is also regulated by an apoptosis-like process. As in Fig. 3, there is some micronuclear loss even in control cells.

**Fig. 5.**
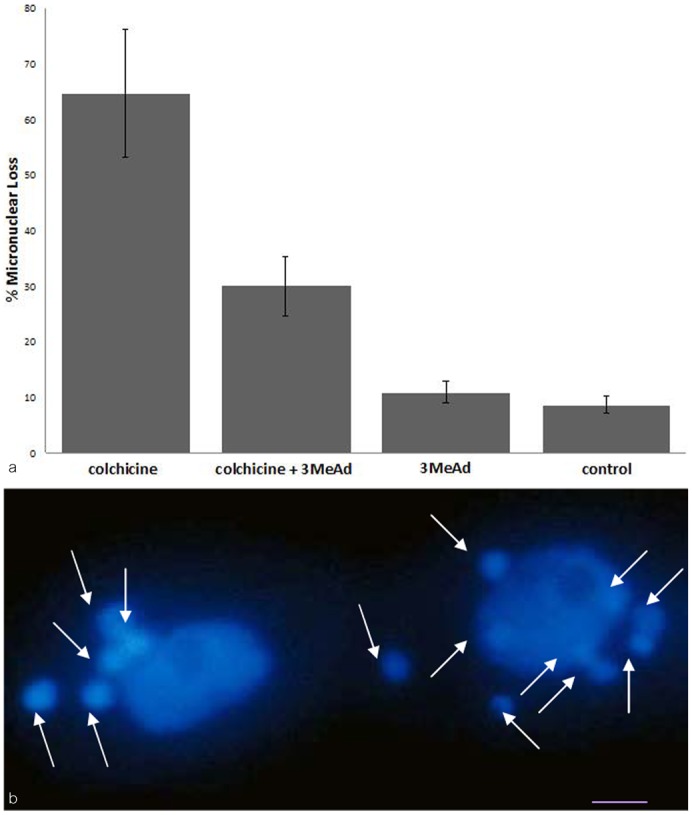
The effect of 3 methyladenine, an inhibitor of autophagy. (a) Bar graph showing the effect of 3 methyladenine, an inhibitor of autophagy, on CIMD. In this set of experiments, about 60% of cells in pairs lost their micronuclei with colchicine (5 mg/ml, 2.5–7.5 hours after mixing mating types). However, when 3 methyladenine was added together with colchicine, micronuclear loss was reduced by about half. Control cells without colchicine, and cells with 3 methyladenine, also show some micronuclear loss. (b) DAPI stained cells after prolonged exposure to 3 methyladenine. 3 Methyladenine (15 mM) was added at 4 hours after mixing mating types, and assayed at 12 hours. In addition to two large macronuclei, which did not condense, nor were they resorbed, 14 smaller nuclei are visible in two cells of a pair (arrows). (Nuclei can shift from one cell to another when large openings in the conjugation junction occur.) This is the predicted result if developmentally programmed resorption of nuclei is prevented. Scale bar: 5 µm.

Autophagy plays an important role in effecting macronuclear elimination (Akematsu et al., 2010). 3 Methyladenine, widely used as an inhibitor of autophagy (Rubinsztein et al., 2007), is effective at 15 mM in reducing the extent of developmentally regulated macronuclear elimination (data not shown). When added together with colchicine to conjugating pairs incubated with colchicine, the loss of micronuclei over a 5 hour period was reduced by ∼50% relative to cells with colchicine alone (Fig. 5). This indicates that autophagy may also play an important role in CIMD.

In controls with 3 methyladenine alone, meiosis is not blocked, and four post-meiotic haploid nuclei are formed. However, the developmentally expected resorption of 3 of the 4 post-meiotic nuclei (Fig. 1c,d) does not occur, leaving 4 nuclei in each cell of a pair; this is consistent with the role of 3 methyladenine in blocking autophagy, and confirms a previous report (Santos et al., 2000). Further, 3 methyladenine does not interfere with subsequent micronuclear divisions; one of the haploid nuclei divides by mitosis to form gamete nuclei, gamete nuclear exchange occurs, and the fertilization nucleus divides mitotically, twice, to produce 4 post-mitotic nuclei (Fig. 1d–h). This results in some conjugant pairs with a total of 14 nuclei (3 post-meiotic haploid nuclei plus 4 post-zygotic diploid nuclei in addition to the 1 macronucleus per cell) (Fig. 5b). In control cells the parental macronucleus condenses (a prelude to its resorption), two of the post-zygotic nuclei enlarge slightly to form new developing macronuclei, while the other two become new micronuclei. With 3 methyladenine the parental macronucleus does not condense, nor is it eliminated. Further, developing macronuclei (Fig. 1m) do not form, suggesting that somehow new macronuclei development is tied to old macronucleus condensation. These observations indicate that 3 methyladenine does not interfere with any of the normal nuclear processes that occur during conjugation other than preventing elimination of nuclei. We take this as evidence that resorption of all three types of nuclei, including that of CIMD, is mediated by autophagy.

**Fig. 6.**
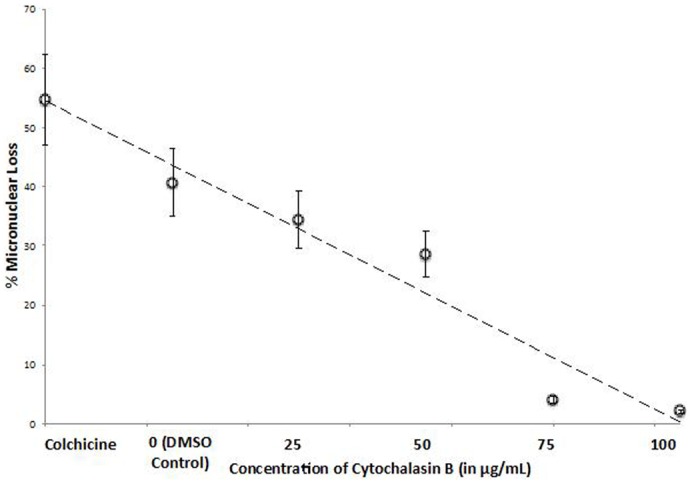
Line graph showing the effect of cytochalasin D, an actin inhibitor, on colchicine-induced micronuclear elimination. Cytochalasin D was solubilized and diluted to different concentrations in 2% DMSO. The graph shows the extent of micronuclear loss with colchicine alone (5 mg/ml, 2.5–7.5 hours after mixing mating types), 2% DMSO alone, and colchicine with cytochalasin in DMSO, at increasing concentrations. The data show that cytochalasin D blocks colchicine-induced micronuclear loss in a concentration-dependent manner. At 100 µg/ml, cytochalasin D almost completely eliminates the effect of colchicine on micronuclear loss. A measurable loss of micronuclei occurs with DMSO alone.

We next co-incubated cell pairs with both colchicine and cytochalasin B, an actin inhibitor. Since selective autophagy depends on actin (Reggiori et al., 2005), we were interested in knowing whether cytochalasin B would interfere with induced micronuclear degeneration. Fig. 6 shows that CIMD is inhibited by cytochalasin B, and in a concentration-dependent manner. These data are consistent with the hypothesis that selective autophagy plays a role in the elimination of micronuclei in colchicine treated conjugants.

**Fig. 7.**
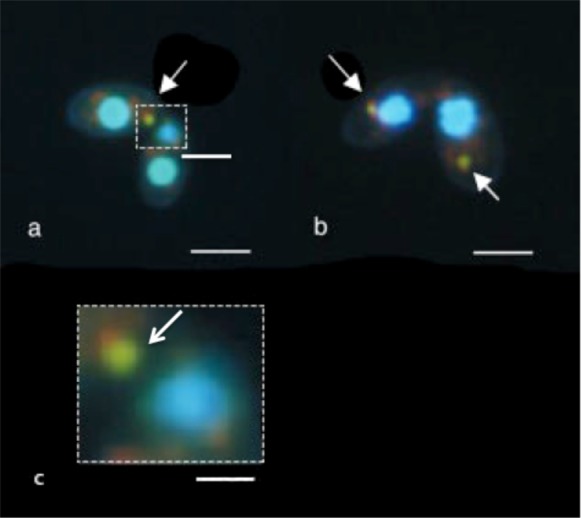
Live pairs double stained with Hoechst 33342 and acridine orange (AO) 3 hours after exposure to colchicine. In all four cells in the two pairs shown in panels a and b, the micronucleus has collapsed. In one cell it is large and blue while in three of the four cells the micronucleus is diminished in size, clearly being resorbed, and is a yellow–green color (arrows), a result of the combination of orange from AO (acidic vesicle), and blue for the Hoechst 33342 (DNA). These data indicate that CIMD occurs in an acidic environment, suggestive of lysosome-dependent autophagy. Panel c shows an enlargement of a portion of panel a as indicated by the dashed-line boxes; the differences in size and staining color of the two types of micronuclei are quite evident. Scale bars: 13.3 µm (a,b), 3.75 µm (c).

Because of the fusion of lysosomes to autophagosomes, acidity of degrading structures is a characteristic of autophagy. Acidification occurs in macronuclear elimination, as detected by staining live cells with the proton-sensitive fluorescent stain, Acridine Orange (AO) together with the DNA-specific Hoechst 33342 (Mpoke and Wolfe, 1997). Acidification also occurs among the three resorbed haploid nuclei (Santos et al., 2000). We therefore expected to see acidified micronuclei among those whose elongation was reversed by colchicine when incubated with the vital fluorescent combination of AO and Hoechst 33342. That was the case; note the yellow–green micronuclei (arrows in Fig. 7a,b) among paired cells exposed to colchicine from 2.5 to 5.5 hours. Stained micronuclei are also smaller than other retracted micronuclei, as expected for structures in the process of being resorbed. The difference between the staining and size of the two kinds of nuclei are strikingly evident in Fig. 7c, an enlargement of a segment from Fig. 7a.

**Fig. 8.**
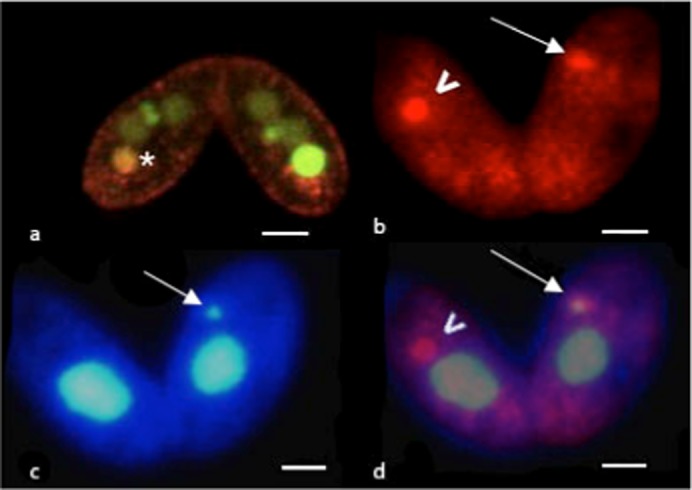
Texas Red Dextran moves from lysosomes into degenerating nuclei. Both cells in the pair in panel a show a single parental macronucleus in the process of degradation. The macronucleus in the left cell is smaller (asterisk), indicating that it is further along in resorption. One can see orange stain in the macronucleus indicating that TRD loaded into lysosome was transferred to the macronucleus, as predicted for autophagy. In panels b and c, cells were incubated in Texas Red Dextran for 1 hour before mixing mating types, and were then exposed to colchicine at 2.5 hours after mixing mating types. Cells were fixed 3 hours later. Concentrated foci of TRD are seen in the posterior ends of a pair of cells in panel b. The same pair is seen in panel c stained with DAPI. The long arrow points to a micronucleus, which cannot be seen in panel b where it is masked by the TRD staining. The arrowhead in panel c points to an aggregate of TRD-containing lysosomes; no micronucleus is visible in the same cell in panel c. Presumably, a micronucleus had been there but is completely resorbed. Panel d is a photographically superimposed image composed of panels b and c, using Photoshop. The small silvery micronucleus in the cell on the right (arrow) is embedded in an aggregate of red TRD-containing lysosomes; in panel b the micronucleus is indistinguishable from the lysosomes indicating that it is loaded with TRD transferred from lysosomes. Scale bars: 7.5 µm (a), 7.2 µm (b–d).

Texas Red Dextran (TRD) is a fluorescently labeled molecule that is resistant to digestion, and as such it is used to label end-stage lysosomes, as well as to monitor autophagy (Kawai et al., 2007), since TRD identifies autophagosome after fusion with lysosomes. This is strikingly manifest in macronuclei whose degeneration is developmentally programmed; see the orange stain inside a degrading macronucleus in the confocal image of Fig. 8a (see asterisk).

If colchicine-induced micronuclear degeneration is achieved by autophagy, then we might expect to see degenerating micronuclei stain with TRD. Fig. 8b shows DAPI staining of a micronucleus (in one cell), while Fig. 8c shows TRD staining of lysosomes in the same fixed pair of cells. In these cells, lysosomes are aggregated. We commonly observe this aggregation when programmed macronuclear degradation is immanent or in process (see the relationship between lysosomes and parental macronucleus in the right cell of Fig. 8a). Co-localization of the micronucleus and lysosomes is clearly seen in Fig. 8d in which the 2 images are photographically superimposed using Photoshop. The micronucleus is also surrounded by TRD-containing lysosomes. It is striking that that in the TRD-stained cell in Fig. 8b, no distinction can be made between micronucleus and lysosomes, suggesting that the micronucleus is infused with TRD. These data indicate that lysosome contents enter some micronuclei, an indication of their being degraded by lysosomes, arguing for autophagy-mediated resorption in CIMD.

In the cell on the left in Fig. 8b there is a strong concentration of TRD staining (arrowhead) without equivalent DAPI staining in Fig. 8c. This may be a residue of TRD left behind from the autophagic digestion of a micronucleus to the point where its DNA is no longer visible with DAPI stain.

We next did a series of experiments to analyze the behavior of cells after colchicine is washed away at 7.5 hours after mixing mating types. First, we examined whether micronuclear elongation would re-occur once the microtubule inhibitor is removed. Samples of cells were fixed at hourly intervals for 5 hours after clearing cells from colchicine. In more than 99% of cells with a micronucleus, at any of the time points, the structure was spherical. We conclude that micronuclear elongation aborted by colchicine is not a reversible process.

Furthermore, there was only a single micronucleus in those cells, ruling out the possibility that meiosis occurs in the absence of micronuclear elongation after colchicine is washed away. This indicates that, under these conditions, meiosis is dependent on prior micronuclear elongation. Under other conditions, e.g. spo11 knockouts, micronuclei blocked from elongation can still undergo meiosis, although aberrantly (Mochizuki et al., 2008).

We next asked about the fate of cells without micronuclei. Cells were washed free of colchicine after the usual 5-hour exposure, and at 24 hours after mixing mating type, the cells were returned to growth medium. At this time, most cells in pairs had separated. At 6 hours afterwards there was a wave of division *among cells with micronuclei* (all dividing cells fixed and stained with DAPI were positive for micronuclei), indicating that they had begun to grow, and their micronuclei were able to undergo mitosis. However, no division figures were observed in cells without micronuclei. These observations indicate that amicronucleate cells are either incapable of growth, or they do so at a rate so slow as to have not been detectable.

We tested the possibility that the absence of normal growth in amicronucleate cells might be related to a failure to take up nutrients from the medium by exposing unpaired cells to particulate India ink for 30 minutes; cells with a functioning oral apparatus can be identified by black food vacuoles. Cells were fixed and stained with DAPI and examined for black vacuoles and blue micronuclei in simultaneous bright field and fluorescence microscopy. Without exception, black vacuoles were not observed among cells that lacked micronuclei, although the great majority of micronucleate cells contained black vacuoles. These observations indicate that without a micronucleus cells lack a functioning phagocytic mechanism and cannot take up food from the medium. That, in turn, could account for their failure to grow and divide.

In *Tetrahymena*, phagocytosis depends on the complex structure of the oral apparatus, with its 4 ciliated membranelles (from which its generic name is derived) and filamentous deep fiber (Nilsson and Williams, 1966; Wolfe, 1970). During the pairing process the oral apparatus is disassembled and then re-assembled after paired cells separate; in cells without a micronucleus it seems that oral apparatus re-assembly is faulty after cell separation. This is in agreement with other experiments demonstrating a dependence of oral apparatus assembly on an existing micronucleus (see Discussion).

## DISCUSSION

We found that, in *Tetrahymena*, micronuclear elongation is of critical importance. When cells are exposed to colchicine, elongation and meiosis are blocked, and the cells cannot progress through conjugation. These observations are in agreement with those reported previously with exposure of conjugants to another microtubule inhibitor, nocodazole, prior to the completion of micronuclear elongation (Wolfe, 1978; Kaczanowski et al., 1985). But, in addition, even after colchicine is washed away from the cells, both elongation and meiosis are still arrested, indicating that the block is irreversible; and conjugation is aborted Those same cells, when returned to growth medium, do divide, and their micronuclei undergo mitosis. Since mitosis, but not meiosis, can occur after colchicine is washed away, there must be some explanation for meiotic failure other than an irreversible impact on microtubule assembly. The simplest possibility is that morphogenetic elongation is a requirement for meiosis.

In many species, a specialized structure, the synaptonemal complex, assists in the alignment of homologous chromosomes. Since this structure is missing in *Tetrahymena* (Wolfe et al., 1976; Loidl and Scherthan, 2004), it was suggested that the process of elongation, by constraining the spatial location of the meiotic chromosomes, might assist in the alignment of chromosomes (Loidl and Scherthan, 2004). That could explain why meiosis fails when elongation is prevented or reversed.

Preventing the formation of double stranded breaks in DNA can also block micronuclear elongation (Mochizuki et al., 2008; Loidl and Mochizuki, 2009). This was shown with the use of a spo11-deletion mutant strain. Spo11 is a protein involved in the formation of double stranded breaks, which in turn are needed for homologous recombination of DNA during meiosis. In these mutants, both elongation and meiosis are blocked. Those data suggest a close relationship between DNA breaks, homologous recombination, the elongation process, and meiosis. Further, by inducing DNA breaks in spo11 mutants, using, for example, ionizing radiation and short-wave UV, micronuclear elongation can be restored (Loidl and Mochizuki, 2009). In that system, meiosis occurs after micronuclear elongation, although not entirely normally. That supports the notion that micronuclear elongation is critical to the success of meiosis. It remains to be determined how micronuclear elongation impacts on chromosomal organization during meiosis.

We also discovered that micronuclei arrested by colchicine are susceptible to complete resorption, a phenomenon referred to here as colchicine-induced micronuclear degeneration. Further, with time, more and more micronuclei are eliminated, reaching 60% by 5 hours after exposure to colchicine. It is likely that the loss would increase even further with time, since a leveling off of the curve of micronuclear loss vs time had not occurred by 5 hours.

The fact that initially, at least, only one cell of a pair (for the most part), lost a micronucleus, suggests that the nuclear loss is a cell autonomous event. If a cytoplasmic signal that could pass through the openings of the conjugation junction were responsible for triggering CIMD, then nuclear resorption would be synchronous in both cells of a pair. Since that does not occur, it is likely that something is acting at the level of the micronucleus itself. In this scenario, pairs with both cells lacking a micronucleus occur as a result of two separate and independent loss events at different times.

It is not obvious what causes micronuclei blocked by colchicine to be resorbed by the cell. Presumably, until resorption has begun, the micronuclei are still capable of DNA replication and mitosis, because cells with non-resorbed micronuclei do grow and divide when returned to growth medium. Therefore, the failure to complete meiosis does not cause irreversible damage to the nucleus. Perhaps the collapsed micronucleus is triggered to undergo resorption because it is not protected by the conjugation junction. As Cole has discussed, nuclear contact with, or proximity to, the conjugation junction may serve as a signal for the survival of one of the four meiotic products (Cole et al., 2008). In the case of the four post-meiotic haploid nuclei, three nuclei are eliminated while one survives; the one that first makes contact with the junction, or migrates to the anterior region of the cell, is the one that survives. In the case reported here, the micronucleus was prevented from dividing or migrating to the conjugation junction. Therefore, it is possible that, like the haploid nuclei that do not migrate to the conjugation junction or anterior region of the cell, it is eliminated.

In *Tetrahymena*, the parental macronucleus is eliminated by a developmentally regulated apoptosis-like process (Davis et al., 1992; Mpoke and Wolfe, 1996; Ejercito and Wolfe, 2003), as are three of the post-meiotic haploid nuclei (Santos et al., 2000); both are controlled by a caspase-dependent activity (Mpoke and Wolfe, 1996; Santos et al., 2000). However, the final elimination of the macronucleus is achieved by autophagy, a lysosome-mediated process (Akematsu et al., 2010). We show here that colchicine-induced micronuclear elimination is also regulated by a caspase-dependent process, and elimination is achieved by autophagy.

Autophagy is generally conceived of as a process that eliminates cytoplasmic material from starving or damaged cells (e.g. Mizushima and Komatsu, 2011). However, at least for programmed elimination of the macronucleus of *Tetrahymena*, the evidence for an autophagic process in its degradation is supported by experiments from several different laboratories. In our laboratory we found that macronuclei in the process of elimination are acidic (Mpoke and Wolfe, 1996), contain lysosomal enzymes (Lu and Wolfe, 2001), and take up Texas Red Dextran from loaded lysosomes (Fig. 8), all indicative of autophagy-mediated nuclear death. Further, genetic analysis supports a role for autophagy in macronuclear elimination during conjugation (Liu and Yao, 2012). Using an inhibitor of autophagy, the phosphoinositide 3 kinase inhibitor, wortmannin, macronuclear degradation in *Tetrahymena* was also prevented (Yakisich and Kapler, 2004). Interestingly, nicotinamide, a sirtuin inhibitor that may impact on autophagy (Zheng et al., 2012), also blocks both macronuclear and micronuclear degradation (Slade et al., 2011), Finally, there is evidence that during macronuclear death the nuclear envelope is transformed converting the macronucleus into an autophagic vesicle to which lysosomes fuse directly (Akematsu et al., 2010).

In this study, we bring evidence that, just as for programmed macronuclear death, both apoptotic regulation and autophagy are necessary for colchicine-induced micronuclear elimination during conjugation. The caspase inhibitor zVAD-fmk blocks colchicine-induced micronuclear elimination, suggesting a role for caspase activity, and therefore apoptosis-like regulation. 3 Methyladenine, a widely used inhibitor of autophagy (Rubinsztein et al., 2007), protects micronuclei from colchicine-induced elimination, providing evidence that autophagy plays a significant role in the process. Cytochalasin b, which disrupts actin polymerization, also interferes with colchicine-induced micronuclear elimination. Since actin is needed for selective autophagy (Reggiori et al., 2005), these data are consistent with a role for selective autophagy in this case. Moreover, the degrading micronuclei are stained by acridine orange in live cells, and mature lysosomes transfer their contents, as assayed by TRD, into degrading micronuclei, both indicative of lysosome-mediated autophagy. It remains to be determined whether micronuclear autophagy occurs by the conventional means, or whether, as in the case of the macronucleus, its nuclear envelope itself converts to an autophagosome (Akematsu et al., 2010).

Nuclear elimination in living cells also occurs in vertebrates during lens cell differentiation (Wride and Sanders, 1998; Wride, 2000; Bassnett, 2009), parasperm nuclear loss in the sea snail, *Ceratostoma foliatum* (Buckland-Nicks and Tompkins, 2005), and skeletal muscle atrophy (Allen et al., 1997) (although recently that observation has been challenged (Bruusgaard et al., 2012)). Also, Gandarillas et al. have observed TUNEL labeled nuclei in keratinocytes of viable cells (Gandarillas et al., 1999), suggesting that nuclear elimination occurs in living cells in skin under certain conditions. As in *Tetrahymena*, in those cases nuclei are not ejected from the cell, but are resorbed. Nuclear loss in *Tetrahymena*, whether the programmed elimination of the parental macronucleus or the colchicine-induced micronuclear elimination reported here, may be a useful model for future research on nuclear degradation in living cells in general.

In addition, this study demonstrates that the potential now exists, in principle, for the controlled selective nuclear elimination of any eukaryotic cell. Exploration of this phenomenon is warranted, since it could become a useful tool for the elimination of certain populations of unwanted cells such as cancer cells. It is interesting to note that extrusion of cancer cell micronuclei can be induced, and that can affect their potential to differentiate (Shimizu et al., 2000).

We observed that micronucleus-deficient cells did not grow, and did not form food vacuoles, when returned to growth medium; this suggests that they were unable to successfully regenerate their oral apparatus and therefore were unable to feed. There is a long history of studies on *Tetrahymena* and *Paramecium* demonstrating a relationship between the micronucleus and the oral apparatus. The gamete nuclei of *Paramecium tetraurelia* were ablated by laser irradiation during autogamy, and this resulted in growth-limited amicronucleate and astomatic cells (Tam and Ng, 1986). In vegetative *Tetrahymena* cells made amicronucleate by laser ablation, or by preventing mitosis while undergoing cell division, lesions in oral apparatus structure and function were observed, and growth was blocked (Wells, 1961; Haremaki et al., 1995). Finally, defective telomeres in micronuclei result in delayed cell division and abnormal oral apparatus morphogenesis (Kirk et al., 2008). So, the results of a number of different experiments indicate that the micronucleus plays some role in oral apparatus reconstruction. At this time, the mechanistic relationship between the micronucleus and the assembly of the oral apparatus remains a puzzle.

But it is interesting that several species of *Tetrahymena*, such as *T. pyriformis*, *T. elliotti*, *T. fergusoni* and *T. lwoffi* (Nanney and McCoy, 1976) are amicronucleate, and all grow quite well. Under limited laboratory conditions, in this study, amicronucleate cells did not succeed in growing. But it may be possible that an occasional cell could arise that loses its micronucleus and may still be able to eat. Such a cell could then grow and divide and would lead to formation of a new species because it could no longer be a party to genetic exchange. If so, that would offer a credible, perhaps testable, explanation for the remarkable appearance of amicronucleate species.
